# Supplementary material for: Cumulative Fluid Balance during Extracorporeal Membrane Oxygenation and Mortality in Patients with Acute Respiratory Distress Syndrome
Source: Membranes (Basel). 2021 Jul 28;11(8):567. doi: 10.3390/membranes11080567 (PMC8402131; doi:10.3390/membranes11080567)
Supplement: Supplementary file 1 [file membranes-11-00567-s001.zip › membranes-1291164-supplementary.pdf]

Table S1. Ventilator settings before and during the first 3 days of ECMO between the earlier years and later years.

Variables	2006-2011 (n = 77)	2012-2015 (n = 75)	*p*
PaO_2/FiO_2 (mm Hg) before ECMO	56 (47-68)	74 (57-107)	0.001
Ventilator settings before ECMO			
MP (J/min)	25.4 ± 9.1	22.1 ± 9.8	0.032
Tidal volume (ml/kg PBW)	7.6 ± 2.4	7.9 ± 2.4	0.535
PEEP (cmH_2O)	12.6 ± 2.7	11.3 ± 2.8	0.004
Peak inspiratory pressure (cm H_2O)	35.7 ± 7.3	32.1 ± 4.9	<0.001
Mean airway pressure (cm H_2O)	20.2 ± 4.3	17.0 ± 3.9	<0.001
Dynamic compliance (ml/cm H_2O)	21.1 ± 9.8	24.1 ± 12.5	0.111
PaO_2/FiO_2 (mm Hg) from day 1 to day 3 on ECMO	165 (126-234)	196 (141-240)	0.094
Ventilator settings from day 1 to day 3 on ECMO			
MP (J/min)	13.7 ± 6.0	10.5 ± 6.1	0.001
Tidal volume (ml/kg PBW)	6.5 ± 2.1	5.5 ± 2.2	0.005
PEEP (cmH_2O)	10.6 ± 2.6	13.4 ± 3.3	<0.001
Peak inspiratory pressure (cm H_2O)	32.9 ± 6.0	30.5 ± 5.0	0.007
Mean airway pressure (cm H_2O)	16.9 ± 3.4	18.5 ± 4.4	0.010
Dynamic compliance (ml/cm H_2O)	18.3 ± 7.4	20.1 ± 8.7	0.168

Data are presented as mean ± standard deviation or median (interquartile range)

Abbreviations: ECMO, extracorporeal membrane oxygenation; FiO_2 fraction of inspired oxygen, MP mechanical power, PaO_2 partial pressure of oxygen in arterial blood, PBW predicted body weight, PEEP positive end-expiratory pressure.
